# Complete mitogenome sequence of *Anopheles coustani* from São Tomé island

**DOI:** 10.1080/23802359.2020.1823273

**Published:** 2020-09-18

**Authors:** Melina Campos, Marc Crepeau, Yoosook Lee, Hans Gripkey, Herodes Rompão, Anthony J. Cornel, João Pinto, Gregory C. Lanzaro

**Affiliations:** a Vector Genetics Laboratory, Department of Pathology, Microbiology and Immunology, UC Davis, Davis, CA, USA; b Florida Medical Entomology Laboratory, University of Florida, Vero Beach, FL, USA; c Programa Nacional de Luta Contra o Paludismo, São Tomé, São Tomé and Príncipe; d Mosquito Control Research Laboratory, Department of Entomology and Nematology, University of California, Parlier, CA, USA; e Global Health and Tropical Medicine, Instituto de Higiene e Medicina Tropical, Universidade Nova de Lisboa, Lisboa, Portugal

**Keywords:** Mosquito, Africa, malaria vector, genome, sequencing

## Abstract

We report the first complete mitogenome (Mt) sequence of *Anopheles coustani,* an understudied malaria vector in Africa. The sequence was extracted from one individual mosquito from São Tomé island. The length of the *A. coustani* Mt genome was 15,408 bp with 79.3% AT content. Phylogenetic analysis revealed that *A. coustani* is most closely related to *A. sinensis* (93.5% of identity); and 90.1% identical to *A. gambiae* complex members.

*Anopheles coustani* was first described by Laveran (1900) from specimens collected in Madagascar. This species is widespread and abundant over much of the African continent (Coetzee 1994). However, this species has been understudied because it is widely considered as a secondary malaria vector. Recent studies have questioned its supposed negligible importance in malaria transmission, since specimens of *A. coustani* were persistently found infected with *Plasmodium* in Cameroon (Antonio-Nkondjio et al. 2006), Kenya (Mwangangi et al. 2013) and Madagascar (Nepomichene et al. 2015; Tedrow et al. 2019). Here, we report the complete mitogenome (Mt) sequence of a field-collected *A. coustani* from São Tomé island (O°22’N 6°42’E). The specimen sequenced was an adult female collected by human landing catch outside of houses in December 2019. *Anopheles coustani* and *A. gambiae s.l* are the only anophelines and potential malaria vectors found on the island (Pinto et al. 2000).

DNA was extracted using a Qiagen Biosprint following our established protocol (Nieman et al. 2015). Library preparation was conducted using 10 ng of genomic DNA as input, as described by Yamasaki et al. (2016). Size selection of the library and clean-up was performed using AMPure SPRI beads (Beckman Coulter Life Sciences, Indianapolis, Indiana, USA). The library was sequenced for 150 bp paired-end reads using a HiSeq 4000 instrument (Illumina, San Diego, CA) at Novogene Corporation. Raw-sequencing reads were used to assemble the Mt contig using NOVOPlasty version 2.6.7 (Dierckxsens et al. 2017).

The length of the *A. coustani* Mt (Genbank: MT806097) is 15,408 bp and the percentage of A + T was 79.3%. The 672 bp long COI fragment spanning 1464-2135 of the *A. coustani* Mt is highly similar to the COI fragment sequences of *A. coustani* from Mali (Huestis et al. 2019), Guinea-Bissau (Gordicho et al. 2014) and Kenya (St Laurent et al. 2016) with an average pairwise identity of 99.4% (±0.2 SD). The 622 bp long COII fragment spanning Mt:3054-3675 was 99.7% identical to the published *A. coustani* COII sequence from Gabon (Ayala et al. 2019). In addition, we sequenced the internal transcribed spacer 2 (ITS2; GenBank: MT791041) region of nuclear ribosomal DNA which was 99.64% identical to the published *A. coustani* from Guinea (Cansado-Utrilla et al. 2020).

A phylogeny including other primary and secondary malaria vector species and *Culex quinquefasciatus* as an outgroup is illustrated in Figure 1. The Jukes-Cantor model was used to calculate pairwise genetic distances and the neighbour-joining method was used to build the phylogenetic tree using Geneious Prime 2020.1.2 (https://www.geneious.com). *Anopheles coustani* was most closely related to the Asian malaria vector, *Anopheles sinensis* (93.5% of identity); and 90.1% on average sequence similarity with African malaria vectors of the *A. gambiae* complex (*A. gambiae s.s.*, *A. coluzzii*, *A. arabiensis*, *A. melas*, *A. merus*). These results are consistent with the formal taxonomic arrangement of these species in which *A. coustani* and *A. sinensis* are in the same subgenus, *Anopheles*, whereas *A. gambiae s.l.* is in the subgenus *Cellia* (Knight and Stone 1977). The extracted DNA sample is maintained in the Vector Genetics Laboratory specimen archive at UC Davis with Accession ID 1219-ST-PGA3-033 and metadata record is available through https://popi.ucdavis.edu/.

**Figure 1.**
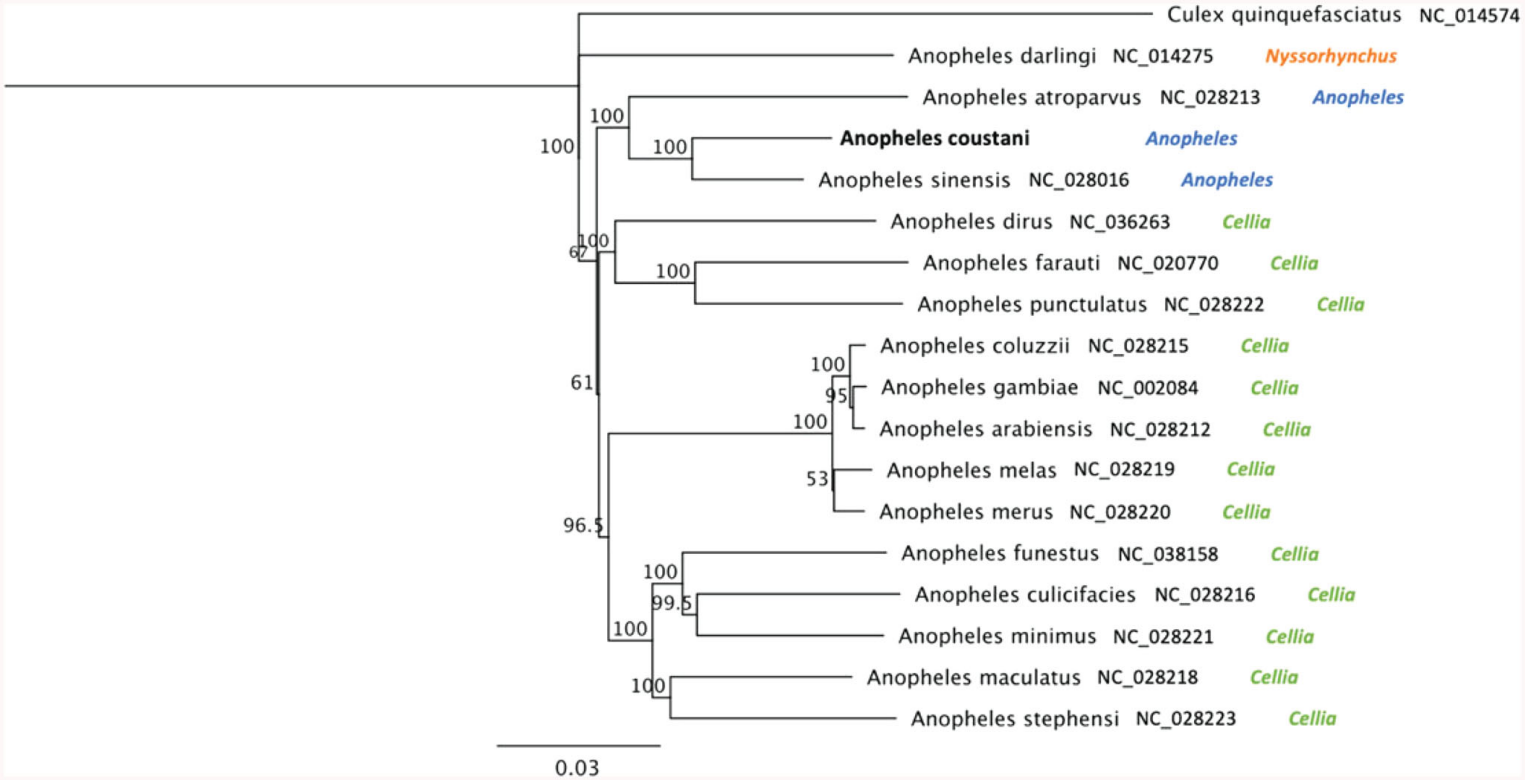
Phylogenetic tree based on mitogenome sequences of anophelines. GenBank IDs and anopheline’ subgenus are provided next to each species name. Numbers at nodes indicate bootstrap values out of 200 replicates. *Culex quinquefasciatus* was considered as an outgroup. Branch length scale bar indicates relative differences (0.03 = 3% nucleotide difference).

The mitogenome was annotated using MITOS (Bernt et al. 2013) using the invertebrate genetic code under default settings. The gene start and end positions and gene orders were by and large consistent with published annotations of mosquito Mt sequences used in the phylogeny. This new annotated mitogenome, from which additional genetic markers may be retrieved, will serve as a basis for subsequent mtDNA-based phylogenetic analyses of this species.

## Data Availability

The data that support the findings of this study are available in Genbank (mitogenome: MT806097; ITS2: MT791041). The meta data associated with it as well as the access to the DNA specimen is available through PopI (https://popi.ucdavis.edu/) with sample ID 1219-ST-PGA3-033.
